# Evaluation of the Urine Albumin-to-Creatinine Ratio (UAC) for Early Renal Disease: A Large-Scale Clinical Study in Cats

**DOI:** 10.3390/ani15142098

**Published:** 2025-07-16

**Authors:** Ye-Eun Cha, Soo-Yeol Lee, Min-Hee Kang, Hyun-Min Kang, Dong-Jae Kang, Hee-Myung Park

**Affiliations:** 1 Department of Veterinary Internal Medicine, College of Veterinary Medicine, Konkuk University, Seoul 05029, Republic of Korea; chaye97@naver.com (Y.-E.C.); dltnduf587@naver.com (S.-Y.L.); wow9315@naver.com (H.-M.K.); rkdehdwo@naver.com (D.-J.K.); 2 Department of Bio-animal Health, Jangan University, Hwaseong 18331, Republic of Korea; mhkang@jangan.ac.kr

**Keywords:** albuminuria, urine albumin-to-creatinine ratio, renal biomarkers, chronic kidney disease, feline

## Abstract

CKD is a leading cause of illness in older cats, but early detection is challenging due to the limitations of conventional biomarkers. This study evaluated the clinical utility of the UAC for identifying early-stage renal dysfunction in cats and proposed a diagnostic matrix incorporating the UAC with other biomarkers. Blood and urine samples from 59 healthy cats and 190 cats with CKD were analyzed, and UAC levels were compared with symmetric dimethylarginine (SDMA), creatinine, and blood urea nitrogen (BUN). UAC values were significantly elevated in cats with CKD, including those in stage 1. Receiver operating characteristic analysis identified a UAC cut-off of 16.3 mg/g, yielding 100% specificity and 43.7% sensitivity for early-stage CKD classification. The UAC showed significant correlations with other renal biomarkers and was incorporated into a multi-parameter matrix to support disease staging. These findings suggest that the UAC may be a promising supplementary biomarker for evaluating renal function in cats and could aid clinical decision-making when interpreted in conjunction with established diagnostic parameters.

## Simple Summary

Chronic kidney disease (CKD) is a common and serious condition in older cats, but it often goes unnoticed in the early stages because the signs are mild or difficult to detect. To help improve early recognition, this study evaluated a simple urine test called the urine albumin-to-creatinine ratio (UAC), which reflects the kidney’s ability to prevent albumin from leaking into the urine. We collected blood and urine samples from healthy cats and from cats at different stages of kidney disease. Cats with kidney disease had more albumin in their urine, including those in the early stages when other test results appeared normal. Based on this, we developed a tool to help veterinarians interpret UAC results alongside other common tests to better evaluate renal function. These findings suggest that measuring albumin in urine may offer a useful addition to existing tools for the early assessment of kidney health in cats, particularly when interpreted alongside other routine tests.

## 1. Introduction

CKD is one of the most prevalent chronic conditions in aging cats, affecting up to 30–40% of cats older than 10 years and accounting for approximately 13–17% of deaths in this population [1,2]. The insidious onset and gradual progression of feline CKD often result in delayed diagnosis, highlighting the need for sensitive and practical diagnostic tools for early-stage assessment and disease monitoring. Traditionally, assessment of renal function has relied on serum creatinine, BUN, and urine-specific gravity (USG), which primarily reflect changes in glomerular filtration rate (GFR) [3]. However, these markers often fail to detect subclinical renal dysfunction, particularly in cats with preserved GFR or structural damage not yet reflected in filtration-based parameters.

In recent years, novel renal biomarkers have been introduced to enhance early detection and provide a more comprehensive evaluation of renal function. Among them, SDMA has been recognized as a reliable surrogate marker for GFR, while the urine protein-to-creatinine ratio (UPC) and albuminuria are used to assess glomerular and tubular injury. Additional experimental biomarkers such as neutrophil gelatinase-associated lipocalin, cystatin C, and kidney injury molecule-1 are under investigation for their roles in tubular damage detection [3]. Despite this progress, the availability, cost, and limited integration of these assays into general veterinary practice remain challenges.

In human medicine, urinary albumin excretion, typically assessed by the UAC, is widely used to detect early kidney damage and to stratify the risk of disease progression, even in individuals with normal GFR [4,5,6,7,8,9]. Albuminuria has been shown to precede measurable changes in GFR and serves as a predictor of renal and cardiovascular outcomes. Although the UAC is not routinely used in veterinary medicine, it has drawn increasing attention as a potential early marker for feline CKD [10,11]. Albumin in urine may reflect glomerular leakage, impaired tubular reabsorption, or both, and thus could provide diagnostic insight complementary to filtration-based markers such as SDMA or creatinine [8,11].

Given the increasing prevalence of CKD in cats and the limitations of current diagnostic tools, there is a need to investigate whether the UAC can serve as a sensitive indicator of early renal dysfunction and offer added clinical value when interpreted alongside other biomarkers. Furthermore, systematic approaches to risk stratification in feline CKD are currently lacking. Inspired by risk matrices developed in human nephrology, a composite assessment model that incorporates the UAC with conventional renal biomarkers may provide a more integrative approach to disease staging and clinical decision-making.

This study aimed to evaluate the clinical utility of the UAC in feline CKD by establishing reference values and assessing its association with established renal markers across IRIS stages. Additionally, we developed a risk assessment model that combines the UAC with conventional renal biomarkers to support early-stage assessment and enhance interpretation of renal function in clinical practice.

## 2. Materials and Methods

### 2.1. Study Design and Case Enrollment

This cross-sectional study was conducted between April and July 2023 across five local animal hospitals in South Korea. A total of 300 paired blood and urine samples were collected from client-owned cats, including both healthy individuals and those previously diagnosed with CKD. Cats with CKD were classified according to the IRIS staging system (2023 modified guidelines) [12], while healthy cats were confirmed through clinical and diagnostic evaluations.

Each animal underwent physical examination, including assessment of hydration status and body condition, along with diagnostic testing. Laboratory evaluation included complete blood counts, serum biochemistry (SDMA, BUN, and creatinine), and urinalysis (USG, UPC, UAC, and urine cytology). Abdominal ultrasonography was performed to evaluate kidney size and morphology, including assessments of echogenicity, corticomedullary distinction, and contour irregularities.

Although systemic hypertension is a known contributor to albuminuria, blood pressure measurements were excluded from the analysis due to variability in equipment, inconsistency in methodology among hospitals, and incomplete records in some cases.

The study protocol was reviewed and approved by the Institutional Animal Care and Use Committee (approval number CPT-23-002-R), and all samples were collected with informed client consent.

### 2.2. Study Population: Inclusion and Exclusion Criteria

A total of 59 healthy cats and 190 cats with CKD were included in the study. Diagnosis of CKD was based on clinical history, serum biochemistry (BUN, creatinine, SDMA), decreased USG, and abnormal kidney size or contour confirmed through palpation and ultrasonography, along with medical records indicating ongoing renal management. Staging of CKD followed the IRIS guidelines (2023 modification): stage 1 (*n* = 22), stage 2 (*n* = 100), stage 3 (*n* = 46), and stage 4 (*n* = 22). Stage 1 CKD was defined by persistent increases in SDMA (≥14 µg/dL on at least two measurements over a minimum interval of 2 weeks), and/or UPC (>0.2), and/or USG (<1.035), in the absence of identifiable non-renal causes. Stages 2 to 4 were classified based on serum creatinine levels: stage 2 (1.6–2.8 mg/dL), stage 3 (2.9–5.0 mg/dL), and stage 4 (>5.0 mg/dL).

Healthy cats were included if they showed no clinical signs of illness and had unremarkable results on physical examination, hematology, biochemistry, urinalysis, and diagnostic imaging (as available), including ultrasonography.

Exclusion criteria were applied to both groups. Cats were excluded if they showed evidence of systemic illness, urinary tract infection, hematuria, crystalluria, bacteriuria, or post-renal proteinuria. Cats with severe dehydration, hyperlipidemia, hemolyzed blood samples, or suspected acute kidney injury (AKI) were also excluded. Urine sediment was examined to rule out inflammatory or abnormal cells. Cases with suspected bacterial cystitis underwent urine culture to confirm absence of infection. Cystocentesis samples showing more than 3 white blood cells per high-power field (hpf) or catheterized samples with more than 5 white blood cells/hpf were excluded to minimize the influence of post-renal inflammation.

To ensure exclusion of AKI, medical records were reviewed for any recent history of dehydration, anuria, nephrotoxic drug exposure, or acute illness. In total, 51 cats were excluded due to evidence of systemic disease, AKI, or pre- or post-renal causes of proteinuria unrelated to CKD.

### 2.3. Laboratory Analysis and Biomarker Measurement

Blood and urine samples were collected via jugular venipuncture and ultrasound-guided cystocentesis. All samples were immediately refrigerated and analyzed within 2 h to minimize pre-analytical variability. Residual samples were stored at −70 °C until batch analysis was performed.

Serum biomarkers, including SDMA, BUN, and creatinine, were measured using a Catalyst One Chemistry Analyzer (IDEXX Inc., Westbrook, ME, USA), following the manufacturer’s instructions. USG was determined using a handheld refractometer (ATAGO Inc., Tokyo, Japan). The UPC was calculated using the same chemistry analyzer.

The UAC was measured using the Care Sign-VTM Analyzer (i-SENS Inc., Seoul, Republic of Korea), which quantifies urinary albumin via immunoturbidimetry and creatinine via chemical colorimetry. The assay demonstrated a coefficient of variation (CV) of 8% for reproducibility and 15% for accuracy across repeated measures, based on internal quality control data. Although not universally standardized across laboratories, the method has shown acceptable performance for clinical use. A 40 µL urine sample was loaded into a single-use cartridge and analyzed within 7 min.

Reference intervals for each biomarker were applied as follows: SDMA < 14 µg/dL, creatinine < 1.6 mg/dL, USG > 1.035, and UPC < 0.2, in accordance with IRIS guidelines. The reference range for BUN (14–36 mg/dL) was based on the manufacturer’s specifications. For the UAC, a reference threshold of 30 mg/g was adopted from a previous study evaluating its prognostic relevance in feline CKD [10]. This value was used as a reference point for evaluating the diagnostic performance of the UAC in this study.

### 2.4. Development of a Risk Matrix for Feline CKD Incorporating the UAC

A composite risk matrix was developed to assess the severity of CKD in cats by integrating the UAC with established renal biomarkers. The matrix design was adapted from the CKD risk classification framework proposed by the Kidney Disease: Improving Global Outcomes (KDIGO) Work Group in human medicine, with adjustments to accommodate feline-specific staging guidelines and biomarker characteristics [4].

The vertical axis of the matrix represents blood-based biomarkers, including creatinine and SDMA, categorized according to the IRIS staging system. The horizontal axis consists of urinary biomarkers, specifically the USG, UPC, and UAC, which were stratified based on the IRIS sub-staging guidelines.

Risk levels within the matrix were determined using data from previous studies on feline CKD progression [10,13,14,15,16,17]. For the UAC, two reference thresholds were adopted from published literature: 30 mg/g, corresponding to the commonly accepted definition of microalbuminuria, and 82 mg/g, representing the upper limit observed in healthy cats [10].

Each biomarker range was converted to a score using a logarithmic transformation, and scores at each axis intersection were summed [18]. The resulting total scores were used to categorize cats into four risk levels, represented by a color-coded system: green (low risk), yellow (moderate risk), orange (moderate to high risk), and red (very high risk).

### 2.5. Statistical Analysis

All statistical analyses were performed using SPSS software (version 28.0; IBM Corp., Armonk, NY, USA). The Kolmogorov–Smirnov test was used to assess the normality of continuous variables. Differences in age and body weight between healthy cats and those with CKD were analyzed using the Student’s *t*-test or Mann–Whitney U test, depending on data distribution. Categorical variables such as sex and breed were compared using the chi-square test or Fisher’s exact test, as appropriate.

Comparisons of renal biomarkers (SDMA, BUN, creatinine, USG, UPC, and UAC) between healthy and CKD groups were performed using the Mann–Whitney U test. Biomarker differences across IRIS stages were assessed using the Kruskal–Wallis test followed by Holm-adjusted post hoc comparisons to control for multiple testing.

Spearman’s rank correlation coefficient was calculated to evaluate the relationships between the UAC and other renal biomarkers. Receiver operating characteristic (ROC) curve analysis was conducted to determine the diagnostic performance and optimal cut-off values for the UAC and SDMA, with the area under the curve (AUC), sensitivity, and specificity reported. Statistical significance was set at *p* < 0.05 for all analyses.

## 3. Results

### 3.1. Characteristics of the Study Population

A total of 249 client-owned cats were included in the study, comprising 59 healthy cats and 190 cats diagnosed with CKD. The demographic and clinical characteristics of each group are summarized in Table 1. Cats in the CKD group were significantly older than those in the healthy group (9.5 ± 4.9 years vs. 6.1 ± 3.8 years, *p* < 0.05), which aligns with the age-related prevalence of CKD in feline populations. However, there was no significant difference in body weight between the two groups (CKD: 4.7 ± 1.4 kg; healthy: 4.7 ± 1.5 kg, *p* > 0.05).

Most cats in both groups were castrated males or spayed females. Korean Shorthairs were the most frequently represented breed across the study population, reflecting the general domestic cat demographics in South Korea.

### 3.2. Comparison of Renal Biomarkers

Renal biomarker values in healthy cats and those with CKD are summarized in Table 2. All measured biomarkers—SDMA, BUN, creatinine, USG, UPC, and UAC—showed statistically significant differences between the two groups (*p* < 0.05). In particular, UAC values were markedly higher in cats with CKD compared to healthy cats.

Table 3 presents biomarker comparisons across CKD stages based on IRIS classification. USG, UPC, and UAC values were significantly different between healthy cats and cats in all CKD stages, including stage 1 (*p* < 0.05). In contrast, BUN and creatinine levels did not show significant differences between healthy cats and those in IRIS stage 1 but were significantly elevated in stages 2 through 4 (*p* < 0.05). Similarly, SDMA concentrations were significantly higher in stages 3 and 4 compared to healthy cats, but not in stage 1.

UAC values increased progressively with advancing IRIS stages, showing the lowest levels in healthy cats and the highest in stage 4 CKD. This trend was consistent with the overall pattern of renal dysfunction severity.

### 3.3. Correlation Between the UAC and Other Renal Biomarkers

**Figure 1:**
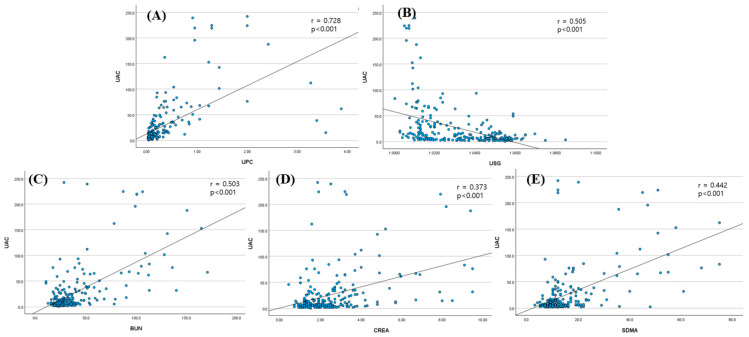
**Spearman correlation plots between the UAC and other renal biomarkers in healthy cats and cats with CKD.** Scatter plots show the relationships between the UAC and (**A**) the UPC, (**B**) USG, (**C**) BUN, (**D**) creatinine, and (**E**) SDMA. The UAC demonstrated a strong positive correlation with the UPC, moderate correlations with BUN and SDMA, a weak correlation with creatinine, and a moderate negative correlation with USG. BUN: blood urea nitrogen; CKD: chronic kidney disease; CREA: creatinine; SDMA: symmetric dimethylarginine; UAC: urine albumin-to-creatinine ratio; UPC: urine protein-to-creatinine ratio; USG: urine-specific gravity.

The relationships between the UAC and other renal biomarkers are shown in Figure 1. The UAC exhibited a strong positive correlation with the UPC (r = 0.728, *p* < 0.05) and a moderate negative correlation with USG (r = –0.505, *p* < 0.05). In addition, the UAC showed moderate positive correlations with BUN (r = 0.503, *p* < 0.05) and SDMA (r = 0.442, *p* < 0.05), and a weak positive correlation with creatinine (r = 0.373, *p* < 0.05).

**Figure 2:**
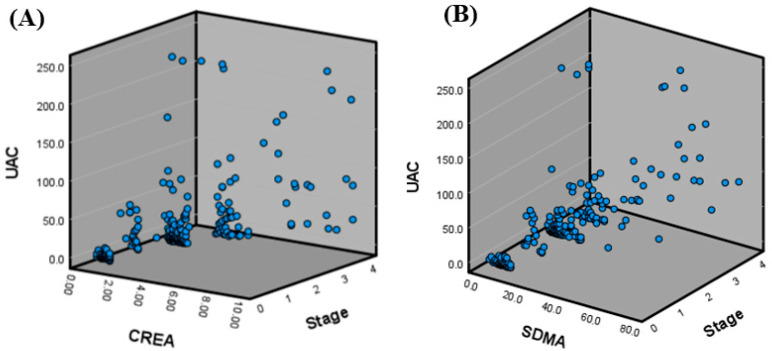
**Three-dimensional scatter plots of UAC, creatinine or SDMA, and CKD IRIS stage in healthy cats and cats with CKD.** (**A**) Relationship between the UAC, creatinine, and CKD IRIS stage. (**B**) Relationship between the UAC, SDMA, and CKD IRIS stage. Cats with similar SDMA or creatinine levels showed a wide range of UAC values across different IRIS stages, indicating that the UAC may provide additional information beyond traditional filtration-based markers. CKD: chronic kidney disease; IRIS: International Renal Interest Society; SDMA: symmetric dimethylarginine; UAC: urine albumin-to-creatinine ratio.

Despite the trend of increasing UAC with advancing CKD, Figure 2 illustrates that UAC values varied considerably among cats with similar creatinine or SDMA levels. This variability was visualized in a three-dimensional scatter plot, where cats with comparable glomerular filtration markers displayed a wide distribution of UAC values.

These findings indicate that the UAC may reflect renal pathological changes, including both glomerular and tubular alterations, that are not fully captured by filtration-based markers such as SDMA or creatinine.

### 3.4. Diagnostic Utility of the UAC and SDMA for Early CKD Detection

ROC curve analysis was conducted to evaluate the utility of the UAC and SDMA in distinguishing cats with early-stage CKD (IRIS stages 1 and 2) from healthy cats. The AUC for the UAC was 0.742 (95% CI: 0.675–0.809, *p* < 0.05), and for SDMA it was 0.717 (95% CI: 0.648–0.785, *p* < 0.05), indicating moderate diagnostic accuracy for both biomarkers, with the UAC slightly outperforming SDMA.

For the UAC, the optimal cutoff value determined by ROC analysis was 16.3 mg/g, which yielded a sensitivity of 100% and a specificity of 43.7%. For SDMA, an empirically derived cutoff of 11.5 µg/dL provided a sensitivity of 58.0% and a specificity of 73.5%. These values were selected based on our study data to ensure a balanced comparison between biomarkers. Notably, the optimal UAC threshold identified in this study was lower than the commonly used reference value of 30 mg/g. Accordingly, a grey zone between 16.3 and 30 mg/g was defined to indicate an intermediate risk of CKD, which may warrant closer monitoring.

### 3.5. Risk Categorization Using a UAC Matrix

**Figure 3:**
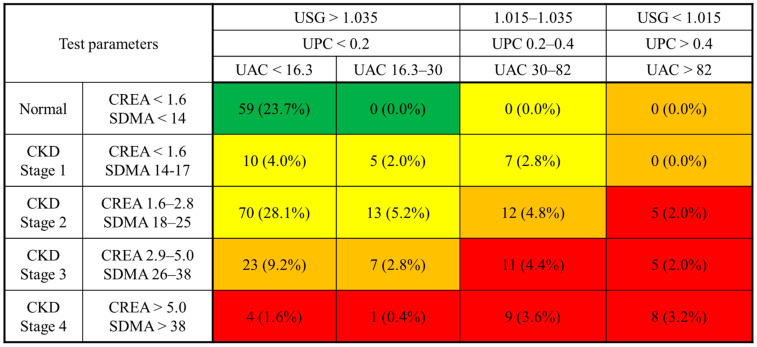
**UAC-based risk stratification matrix for feline CKD using combined categories of USG, UPC, creatinine, and SDMA.** The matrix categorizes risk levels based on UAC ranges (<16.3, 16.3–30, 30–82, >82 mg/g) combined with USG, the UPC, and serum biomarkers (creatinine and SDMA) stratified according to IRIS stages. Each cell shows the number and percentage of cats in the corresponding risk zone, color-coded as green (low risk), yellow (moderate), orange (moderate to high), and red (very high risk). CKD: chronic kidney disease; CREA: creatinine; SDMA: symmetric dimethylarginine; UAC: urine albumin-to-creatinine ratio; UPC: urine protein-to-creatinine ratio; USG: urine-specific gravity.

To support clinical interpretation of UAC values in feline CKD, we developed a risk stratification matrix incorporating the UAC alongside established renal biomarkers, including creatinine and SDMA. Based on UAC thresholds identified in this study, cats were categorized into three risk levels: low risk (<16.3 mg/g), intermediate risk or “grey zone” (16.3–30 mg/g), and high risk (≥30 mg/g). The distribution of cats across these categories, stratified by IRIS stage and other renal biomarkers, is presented in Figure 3.

**Figure 4:**
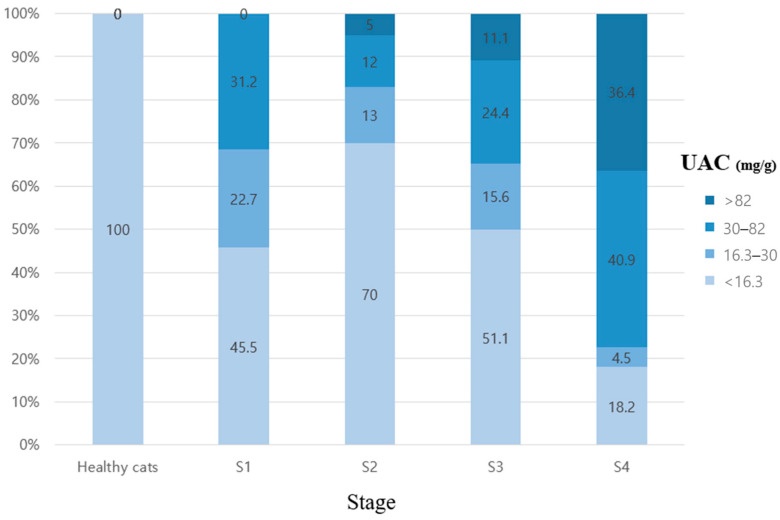
**Stacked bar chart showing the distribution of cats by UAC categories across CKD IRIS stages and healthy controls.** Cats were classified into four UAC categories based on clinically relevant thresholds: <16.3 mg/g, 16.3–30 mg/g (grey zone), 30–82 mg/g, and >82 mg/g. The proportion of cats with an elevated UAC increased progressively with advancing CKD stage, while nearly all healthy cats had UAC values below 16.3 mg/g. This distribution supports the role of the UAC as a staging and risk stratification marker. UAC: urine albumin-to-creatinine ratio; S1–S4: CKD IRIS stages 1–4.

This matrix is further visualized in Figure 4, which illustrates how individual cats clustered within the risk categories according to their UAC, creatinine, and SDMA levels. Among cats in the grey zone, several exhibited elevated SDMA or borderline creatinine values despite being classified as IRIS stage 1 or appearing clinically normal, suggesting potential subclinical renal dysfunction. Conversely, cats in the low-risk category typically showed normal findings across other markers, reinforcing the negative predictive value of the UAC in these cases.

This matrix-based approach provides a structured framework for integrating proteinuria with conventional renal markers. It may aid clinicians in identifying early or borderline renal dysfunction that may not yet meet criteria for overt CKD, facilitating earlier intervention and more tailored monitoring strategies.

## 4. Discussion

This study evaluated the clinical utility of the UAC as an early biomarker for feline CKD and proposed a structured risk matrix integrating the UAC with established renal biomarkers. UAC levels were significantly higher in cats with CKD than in healthy controls and increased progressively across IRIS stages. Notably, the UAC was elevated even in IRIS stage 1 cats, suggesting its utility in detecting early renal pathology before marked GFR decline. However, the UAC did not increase from stage 1 to 2; the mean was slightly lower in stage 2. This non-linear pattern implies that UAC elevation may precede GFR changes but does not progress consistently across stages.

Importantly, many of the stage 1 cats in our study did not exhibit clinical signs or azotemia, suggesting that the UAC may serve as a sensitive marker of subclinical renal injury. This finding distinguishes our results from previous studies that focused on albuminuria primarily in azotemic or symptomatic CKD cases, and supports the utility of the UAC in the early detection of renal dysfunction in otherwise asymptomatic cats.

Consistent with previous studies [1,2,3], CKD was more prevalent in older cats, and mean age differed significantly between healthy and CKD groups, reinforcing the need for early screening in aging feline populations. Although age-related physiological changes may potentially influence renal biomarkers, previous studies have shown that age alone does not significantly affect the UAC, the UPC, or SDMA in healthy cats, suggesting that the differences observed in this study are more likely attributable to CKD status rather than age [19]. Nonetheless, the age imbalance is acknowledged as a limitation, and future studies with age-matched control populations are warranted to further validate these findings. Although body weight can decline in advanced CKD stages [2,20], no significant differences were observed in our study, possibly due to the larger proportion of early- to mid-stage CKD cases.

Multiple renal biomarkers, including SDMA, BUN, creatinine, USG, and the UPC, differed significantly between groups, reflecting functional and structural renal changes. The UAC and the UPC reflect proteinuria from glomerular or tubular damage, whereas SDMA, BUN, and creatinine indicate reduced GFR [3,16,17,21,22]. Significant differences in the UAC, the UPC, and USG in stage 1 cats support their utility as early indicators of renal impairment. However, the staging criteria for IRIS stage 1 include an elevated UPC and reduced USG, which may partially explain the elevated UAC in this group. Future research using histopathology or biomarkers independent of staging definitions may help clarify this relationship. Moreover, SDMA and creatinine represent different aspects of renal function and may be discordant, especially in older cats. Some geriatric cats show elevated SDMA despite normal creatinine levels, suggesting subclinical renal dysfunction not captured by creatinine alone [16]. Evaluating whether these cats also have an increased UAC could help detect early renal injury. Longitudinal studies linking such biomarker profiles with outcomes like survival and quality of life may clarify the prognostic utility of the UAC in feline CKD.

ROC analysis identified a UAC cutoff of 16.3 mg/g for distinguishing CKD cats from healthy ones, yielding 100% specificity but limited sensitivity (43.7%). Given this trade-off, the threshold is best interpreted as a highly specific rule-in value, confirming renal injury when albuminuria is detected, rather than serving as a sensitive screening tool. While the limited sensitivity (43.7%) restricts its utility as a screening tool for early-stage CKD, the high specificity may support its complementary use in confirming renal involvement when albuminuria is present, particularly when interpreted alongside other clinical and biochemical indicators. This threshold is notably lower than the conventional 30 mg/g cutoff cited in previous veterinary studies [10] and more closely aligns with the microalbuminuria range recognized in human nephrology [5,7,8,9,23]. Based on these findings, we proposed a “grey zone” of 16.3–30 mg/g to help identify cats with subclinical or early renal dysfunction. This range may indicate mild renal injury not yet reflected in conventional GFR-based markers such as SDMA or creatinine. Many cats in IRIS stages 1 and 2 fell within this range, underscoring the potential value of the UAC in early detection. For cats with UAC values in the grey zone, we recommend close clinical monitoring, including repeat UAC measurement, serial assessment of other renal biomarkers, blood pressure monitoring, and abdominal ultrasound where feasible. Such follow-up may enable earlier detection of disease progression and more timely therapeutic intervention.

The proposed UAC-based stratification model integrates creatinine, SDMA, USG, and the UPC to enhance renal risk assessment. Visualization of this model (Figure 3) and UAC distribution by stage (Figure 4) demonstrated that higher UAC values were associated with more advanced CKD. Additionally, cats with similar SDMA or creatinine levels exhibited varying degrees of albuminuria, as shown in the 3D scatter plot, suggesting that the UAC captures renal alterations not solely explained by glomerular filtration and may also reflect injury associated with impaired tubular reabsorption. This multifactorial interpretation aligns with previous findings that albuminuria in cats can result from both increased glomerular permeability and reduced tubular reabsorption.

In human medicine, risk matrices combining albuminuria and GFR are widely used to stratify CKD risk and guide treatment decisions [4]. Inspired by this approach, we proposed a feline-specific risk matrix incorporating the UAC with conventional renal biomarkers. UAC levels were significantly higher in CKD cats compared to healthy controls, and elevated even in IRIS stage 1, emphasizing its potential as a sensitive marker of early renal injury. However, there was no significant increase between stage 1 and 2, with the mean UAC in stage 2 being slightly lower than in stage 1. This non-linear trend highlights that while the UAC may detect early renal injury, it does not necessarily increase proportionally across all CKD stages. Moreover, variability in UAC among cats with similar SDMA or creatinine levels suggests that albuminuria provides complementary insight into renal dysfunction, consistent with the multifactorial nature of albuminuria, which may arise from both glomerular leakage and tubular dysfunction. While human risk matrices are often based on longitudinal mortality outcomes, the matrix in this study relied on biomarker-defined risk categories from prior veterinary research [10,13,14,15,16,17,24], as survival data were not available in this cross-sectional cohort.

The prognostic significance of albuminuria is further supported by previous studies. Syme et al. [10] reported hazard ratios for death or euthanasia of 2.4 and 4.9 in cats with UAC levels of 30–82 mg/g and >82 mg/g, respectively. Our study’s lower diagnostic threshold of 16.3 mg/g may facilitate earlier identification of renal injury. However, methodological differences limit direct comparison: Syme et al. used sandwich ELISA, while this study used immunoturbidimetry. These methodological variations may explain discrepancies in UAC reference values between studies. Although the immunoturbidimetric method used in this study demonstrated acceptable reproducibility and accuracy, its comparability with ELISA-based results remains limited. Standardization of assay platforms and calibration across methods is needed for broader clinical application and consistent interpretation of UAC levels in feline CKD. Furthermore, Syme et al. found that hypertension significantly influenced albuminuria, with hypertensive cats having higher UAC values within the same IRIS stage. In our study, blood pressure data were excluded due to inconsistent measurement techniques and incomplete records, despite the established influence of hypertension on albuminuria. This limitation is acknowledged and discussed to emphasize the potential role of hypertension as a confounding variable. Although albuminuria can be influenced by systemic hypertension, the exclusion was necessary to maintain data consistency across multi-center datasets. Future prospective studies with standardized blood pressure assessment should explore the interaction between hypertension and albuminuria in feline CKD.

It is also notable that the UPC demonstrated slightly superior diagnostic accuracy compared to the UAC, with AUC values of 0.876 and 0.742, respectively. In IRIS stage 1, the UPC again showed a higher AUC (0.917 vs. 0.816). This may be due to the small sample size (*n* = 22) or the high UPC values in a subset of cats (*n* = 9/22). In humans, the UAC often increases earlier than the UPC due to early glomerular damage, whereas in cats, glomerular involvement may occur later in the disease process [1]. This species difference may explain why the UAC did not outperform the UPC in our early-stage feline CKD cohort.

This study had several limitations. The relatively small number of cats in IRIS stages 1 and 4 may have limited the statistical power of subgroup analyses and reduced the generalizability of findings in those groups. Additionally, the lack of age-matching between groups and the exclusion of blood pressure measurements are noted limitations that may have influenced the interpretation of UAC levels. Future prospective studies should incorporate standardized blood pressure assessment protocols to better evaluate the interaction between systemic hypertension and albuminuria, especially in early-stage or borderline CKD cases where diagnostic uncertainty may be high. The cross-sectional nature of the study also prevented assessment of prognostic outcomes or longitudinal progression. Lastly, non-standardized UAC measurement methods limit inter-study comparability, underscoring the need for universal assay validation and reference thresholds. Furthermore, this study did not include tubular injury biomarkers such as neutrophil gelatinase-associated lipocalin (NGAL), kidney injury molecule-1 (KIM-1), or N-acetyl-β-D-glucosaminidase (NAG), which could have helped differentiate the underlying source of albuminuria. This limitation was due to the retrospective design and absence of these data in routine clinical records. Future prospective studies incorporating tubular-specific markers would provide a more detailed understanding of the renal injury profile in feline CKD.

Despite these limitations, this study provides clinically meaningful insight into the use of the UAC as an early biomarker for CKD in cats. By identifying a lower diagnostic cutoff and proposing a stratification model integrating the UAC with conventional renal biomarkers, this study provides a valuable tool for identifying cats at risk of CKD progression. The proposed matrix may help general practitioners assess renal risk even in cats with borderline or equivocal laboratory findings. This work contributes to ongoing efforts to improve early diagnosis and individualized management of feline CKD.

## 5. Conclusions

This study suggests the potential clinical utility of the UAC as an early indicator of renal dysfunction in cats. UAC values were characterized in both healthy and CKD-affected cats, and a lower diagnostic threshold was proposed to support earlier recognition of renal impairment. By integrating the UAC with conventional renal biomarkers, a structured framework for assessing disease severity was developed. These findings support the potential role of the UAC as a supplementary marker in multi-parameter assessments for the early detection and management of feline CKD.

## Figures and Tables

**Table 1:** Baseline characteristics of the study population.

Characteristic	Healthy Cats (*n* = 59)	Cats with CKD (*n* = 190)
Age * (years)	6.1 ± 3.8	9.5 ± 4.9
Body weight (kg)	4.7 ± 1.5	4.7 ± 1.4
Sex (*n*)		
Intact male	0	8
Castrated male	34	101
Intact female	7	3
Spayed female	18	78
Breed (*n*)		
Korean Shorthair	29	66
Others ^a^	30	124

Values are presented as mean ± standard deviation or number of cats (*n*). * Significantly different between groups (*p* < 0.05). ^a^ Others include mixed-breed cats and other purebred breeds such as Persian and Russian Blue. CKD: chronic kidney disease.

**Table 2:** Renal biomarker values in healthy cats and cats with CKD.

Variables	Healthy Cats		Cats with CKD		Reference Range	*p*
	Mean ± SD	*n*	Mean ± SD	*n*		
BUN (mg/dL)	23.6 ± 4.4	59	41.9 ± 30.5	190	14–36	<0.05 *
Creatinine (mg/dL)	1.2 ± 0.2	59	2.9 ± 1.7	190	<1.6	<0.05 *
USG	1.050 ± 0.010	52	1.030 ± 0.020	188	>1.035	<0.05 *
UPC	0.04 ± 0.02	59	0.36 ± 0.62	188	<0.2	<0.05 *
SDMA (µg/dL)	9.7 ± 2.6	49	17.1 ± 13.0	176	<14	<0.05 *
UAC (mg/g)	5.5 ± 3.9	59	32.5 ± 48.8	189	<30	<0.05 *

Values are presented as mean ± standard deviation (SD) or number of cats (*n*). BUN: blood urea nitrogen; SDMA: symmetric dimethylarginine; UAC: urine albumin-to-creatinine ratio; UPC: urine protein-to-creatinine ratio; USG: urine-specific gravity. * Statistical significance: *p* < 0.05.

**Table 3:** Renal biomarker values in healthy cats and cats with CKD by IRIS stage.

Variables	Healthy Cats		CKD Stage1		CKD Stage 2		CKD Stage 3		CKD Stage 4	
	Mean ± SD	*n*	Mean ± SD	*n*	Mean ± SD	*n*	Mean ± SD	*n*	Mean ± SD	*n*
BUN (mg/dL)	23.6 ± 4.4	59	23.1 ± 7.0	22	29.7 ± 13.2	100	48.6 ± 22.6	46	101.9 ± 38.6	22
Creatinine (mg/dL)	1.2 ± 0.2	59	1.2 ± 0.3	22	2.1 ± 0.4	100	3.4 ± 0.5	46	6.9 ± 1.7	22
USG	1.050 ± 0.010	52	1.040 ± 0.020	21	1.030 ± 0.010	99	1.020 ± 0.010	46	1.010 ± 0.000	22
UPC	0.04 ± 0.02	59	0.20 ± 0.18	21	0.17 ± 0.30	99	0.37 ± 0.40	46	1.34 ± 1.18	22
SDMA (µg/dL)	9.5 ± 2.5	49	16.9 ± 8.2	16	11.3 ± 4.4	95	15.4 ± 5.3	42	40.3 ± 19.4	22
UAC (mg/g)	5.5 ± 3.9	59	24.2 ± 18.0	21	21.3 ± 39.5	100	38.0 ± 55.4	46	80.4 ± 63.4	22

Values are presented as mean ± standard deviation (SD) or number of cats (*n*). BUN: blood urea nitrogen; SDMA: symmetric dimethylarginine; UAC: urine albumin-to-creatinine ratio; UPC: urine protein-to-creatinine ratio; USG: urine-specific gravity.

## Data Availability

The datasets generated and analyzed during the current study are available from the corresponding author upon reasonable request.
